# Extraction of chemical–protein interactions from the literature using neural networks and narrow instance representation

**DOI:** 10.1093/database/baz095

**Published:** 2019-10-17

**Authors:** Rui Antunes, Sérgio Matos

**Affiliations:** Department of Electronics, Telecommunications and Informatics (DETI), Institute of Electronics and Informatics Engineering of Aveiro (IEETA), University of Aveiro, Aveiro, Portugal

## Abstract

The scientific literature contains large amounts of information on genes, proteins, chemicals and their interactions. Extraction and integration of this information in curated knowledge bases help researchers support their experimental results, leading to new hypotheses and discoveries. This is especially relevant for precision medicine, which aims to understand the individual variability across patient groups in order to select the most appropriate treatments. Methods for improved retrieval and automatic relation extraction from biomedical literature are therefore required for collecting structured information from the growing number of published works. In this paper, we follow a deep learning approach for extracting mentions of chemical–protein interactions from biomedical articles, based on various enhancements over our participation in the BioCreative VI CHEMPROT task. A significant aspect of our best method is the use of a simple deep learning model together with a very narrow representation of the relation instances, using only up to 10 words from the shortest dependency path and the respective dependency edges. Bidirectional long short-term memory recurrent networks or convolutional neural networks are used to build the deep learning models. We report the results of several experiments and show that our best model is competitive with more complex sentence representations or network structures, achieving an F1-score of 0.6306 on the test set. The source code of our work, along with detailed statistics, is publicly available.

## Introduction

As the knowledge of how biological systems work at different structural levels grows, more possibilities arise for applying it in diagnosing and treating common and complex diseases. Furthermore, exploiting the large amounts of biomolecular data from -omics studies and patient-level information recorded in electronic health records offers prospects for precision and personalized medicine [1]. Nonetheless, relevant fine-grained information is constantly being communicated in the form of natural language through scientific publications. To exploit this source of updated knowledge, several methods have been proposed for retrieving relevant articles for database curation [2], and for extracting from the unstructured texts information such as entity mentions [3, 4], biomolecular interactions and events [5, 6] or the clinical and pharmacological impact of genetic mutations [7]. These methods have proven essential for collecting the most recent research results and for expediting database curation [8].

**Figure 1:**
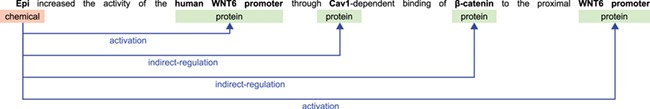
Example sentence illustrating biochemical entities and their relations.

The BioCreative VI CHEMPROT challenge stimulated the development of systems for extracting interactions between chemical compounds (drugs) and GPROs (gene and protein related objects) from running text, given their importance for precision medicine, drug discovery and basic biomedical research [9]. An example illustrating various relations that can be extracted from a single sentence in a publication is shown in Figure 1. The development of systems able to automatically extract such relations may expedite curation work and contribute to the amount of information available in structured annotation databases, in a form that is easily searched and retrieved by researchers.

Data for the CHEMPROT task was composed of PubMed abstracts in which gold-standard entities were provided, and the aim was to detect chemical–protein pairs that expressed a certain interaction. Therefore, the biomedical named entity recognition (NER) step was out of the scope of this task. The organizers defined 10 groups of chemical–protein relations (CPRs) that shared some underlying biological properties, in which only five of them (up-regulation, down-regulation, agonist, antagonist and substrate) were used for evaluation purposes. More detail about the data is presented in Section 3.

This paper describes our participation in the CHEMPROT task together with the improvements we performed after the challenge. At the time of the official evaluation, our system [10] was based on the application of bidirectional long short-term memory (BiLSTM) recurrent neural networks RNNs using features from tokenization, part-of-speech (PoS) tagging and dependency parsing. After the challenge we ran additional experiments including other resources and methods, which allowed the system to perform better. Despite the main idea of our system remaining the same, the final results showed that our adjustments to the system led to an improvement in F1-score of 11 percentage points on the test set. These experiments included adapting the network structure, employing other networks such as convolutional neural networks (CNNs), performing a more meticulous pre-processing, balancing precision and recall, adding more training data from an external repository and testing other pre-trained word embeddings.

This paper is organized as follows: related work is presented in the next section, followed by resources and methods we employed; next, we present our results and discuss possible limitations of our approach; finally, some conclusions and future work directions are given in the last section.

## Related work

Previous research on biomedical relation extraction focused on protein–protein interactions (PPIs) [6] and relations between drugs, genes and diseases [8, 11]. Machine learning methods combined with kernel functions to calculate similarities between instances given some representation were shown to achieve good results in textual relation extraction.

As opposed to the traditional machine learning methods employed in initial works, deep learning techniques eliminate the need for feature engineering, instead using multiple data transformation layers that apply simple non-linear functions to obtain different levels of representation of the input data, intrinsically learning complex classification functions [12]. These strengths have brought much attention with significant successes in several natural language processing tasks, including word sense disambiguation (WSD) [13], text classification [14, 15] and NER [16, 17].

Several works have demonstrated the use of deep neural networks for biomedical relation extraction and classification. For example, Nguyen *et al.* [18] used a CNN with pre-trained word embeddings, outperforming previous state-of-the-art systems for relation classification. Nonetheless, the sequential nature of natural texts can be better modeled by recurrent networks, which contain a feedback loop that allows the network to use information regarding the previous state. LSTM networks are a special type of RNNs in which a set of information gates is introduced in the processing unit that allow these networks to memorize long-term dependencies while avoiding the vanishing gradient problem. Wang *et al.* [19] used BiLSTM networks and features from the dependency structure of the sentences obtaining an F1-score of 0.720 in the DDIExtraction 2013 corpus. Zhang *et al.* [20] also used BiLSTM models for extracting drug–drug interactions (DDIs) achieving a state-of-the-art F1-score of 0.729 in the same dataset. They integrated the shortest dependency path (SDP) and sentence sequences, and concatenated word, PoS and position embeddings into a unique embedding, and an attention mechanism was employed to give more weight to more relevant words.

Methods for extracting chemical–disease relations were evaluated in the BioCreative V CDR task, in which participants were required to identify disease and chemical entities and relations between them [21]. Using the provided gold-standard entities, Zhou *et al.* [22] achieved an F1-score of 0.560 with a hybrid system consisting of a feature-based support vector machine (SVM) model, a tree kernel-based model using dependency features and a LSTM network to generate semantic representations. This result was improved to 0.613 by inclusion of post-processing rules. The same result was achieved by Gu *et al.* [23], also with a hybrid system combining a maximum entropy model with linguistic features, a CNN using dependency parsing information and heuristic rules.

Regarding CPR extraction, the state-of-the-art results were achieved by teams participating in the BioCreative VI CHEMPROT challenge [9], with some improvements described in follow-up works. The best participating team achieved an F1-score of 0.641 using a stacking ensemble combining an SVM, a CNN and a BiLSTM [24, 25]. Lemmatization, PoS and chunk labels from the surrounding entity mentions and from the SDP were used as features for the SVM classifier. For the CNN and BiLSTM, the sentence and shortest path sequences were used, where each word was represented by a concatenation of several embeddings (PoS tags, dependencies, named entities and others). Corbett *et al.* [26] achieved an F1-score of 0.614 using pre-trained word embeddings and a network model with multiple LSTM layers, with the ChemListem NER system used for tokenization [27]. This result was improved to an F1-score of 0.626 in post-challenge experiments [28]. Mehryary *et al.* [29] proposed two different systems: an SVM classifier and an ensemble of neural networks that use LSTM layers. Both systems took features from the dependency parsing graph, although the SVM required more feature engineering. They combined the predictions of the two systems, yet the SVM alone produced the best F1-score (0.610). After the challenge they achieved an F1-score of 0.631 by using their improved artificial neural network (ANN) [30]. Lim *et al.* [31] used ensembles of tree-LSTM networks, achieving an F-score of 0.585 during the challenge. They later improved this result to 0.637 with a revised pre-processing and by using more members in the ensemble, and equaled the best challenge F1-score (0.641) using a shift-reduce parser based network architecture [32]. Lung *et al.* [33, 34] achieved an F1-score of 0.567 using traditional machine learning. Neural networks with attention mechanisms were also followed by Liu *et al.* [35, 36] and Verga *et al.* [37], but achieved lower results. However, the use of attention layers [38, 39] has been shown to be effective in different information extraction tasks such as document classification [40] and relation extraction [41], being an interesting direction to explore.

Zhang and Lu [42] present a semi-supervised approach based on a variational autoencoder for biomedical relation extraction. They evaluated their method in the CHEMPROT dataset experimenting with different number of labeled samples, showing that adding unlabeled data improves the relation extraction mainly when there are only a few hundred training samples. Using 4000 (from a total of 25 071) labeled training instances together with unlabeled data taken from the remaining training instances (with true labels removed), their semi-supervised method achieved an F-score of 0.509.

Lastly, a recent work by Zhang *et al.* [43] achieved the state-of-the-art F-score of 0.659 using BiLSTM models with deep context representation (providing superior sentence representation compared to traditional word embeddings) and multihead attention.

## Materials and methods

This section describes the resources used, the evaluation metric employed and the methods implemented.

### Dataset

The CHEMPROT corpus was created by the BioCreative VI organizers [9], being composed of three distinct sets: training, development and test (Table 1). During the challenge, to hinder manual corrections and to ensure that systems could annotate larger datasets, the organizers included 2599 extra documents in the test set, which were not used for evaluation.

**Table 1:** CHEMPROT dataset statistics

		**Training**	**Development**	**Test**
**Abstracts**	Total	1020	612	800
	With any relation	767	443	620
	With evaluated relations	635	376	514
**Entities**	Chemical	13 017	8004	10 810
	Protein	12 735	7563	10 018
	Total	6437	3558	5744
	Activation (CPR:3)	768	550	665
	Inhibition (CPR:4)	2254	1094	1661
**Relations**	Agonist (CPR:5)	173	116	195
	Antagonist (CPR:6)	235	199	293
	Substrate (CPR:9)	727	457	644

Each document, containing the title and the abstract of a PubMed article, was annotated by expert curators with chemical, protein entity mentions and their relations. The annotation guidelines considered 10 groups of biological interactions, which were designated as CPR groups. However, for this task, only five classes were considered for evaluation purposes: activation (CPR:3), inhibition (CPR:4), agonist (CPR:5), antagonist (CPR:6) and substrate (CPR:9). Table 1 presents detailed dataset statistics.

One can see from Table 1 that not all abstracts contain annotated relations, although all abstracts were annotated with entity mentions. Nevertheless, abstracts without evaluated relations are useful as they can be used to create negative instances for training the system. Only 1525 documents of 2432 (63%) are annotated with evaluated relations. This suggests that it could be a reasonable idea to first apply a document triage step to ignore documents that probably are not relevant for extracting chemical–protein interactions (CPIs), reducing the number of false positive relations, while still considering them for generating negative instances to feed the deep learning model. Though, we did not follow this possibility leaving it as possible future work. Similar binary approaches were followed by Lung *et al.* [33, 34] and Warikoo *et al.* [44] who start by predicting if a CPR pair is positive.

A more scrupulous analysis of the corpus shows that there are some relations between overlapped entities (for example, a protein entity containing a chemical entity), as well as some cross-sentence relations. However, cross-sentence relations appear in a very small number and were deliberately discarded. Also, despite some CHEMPROT relations were classified with more than one CPR group we considered only one label, since these are rare, simplifying the task as a multi-class problem.

### Performance evaluation

The BioCreative VI CHEMPROT organizers considered the micro-averaged precision, recall and balanced micro F1-score for evaluation purposes [9]. Micro F1-score was the official metric used to evaluate and compare the teams’ submissions. This metric was integrated in our pipeline, for measuring the neural network performance at each training epoch, allowing to develop and select the best model dynamically for this specific task.

### Pre-processing

We pre-processed the entire CHEMPROT dataset using the Turku Event Extraction System (TEES) [45] applying a pipeline composed with the GENIA sentence splitter [46], the BLLIP parser [47] using the McClosky and Charniak biomedical parsing model [48] and the Stanford dependency parser [49] (version 3.8.0, released on 2017-06-09). This pre-processing performs sentence splitting, tokenization, PoS tagging and dependency parsing. Sentence splitting is required to obtain all the chemical–protein pair candidates in the same sentence, since these are the only ones we considered. The yielded tokens, PoS tags and dependency labels are encoded using embedding vectors (more detail in the next sections). The dependency parsing structure is also used to find the SDP between the two entities, since previous work had already proven its value for relation extraction [50].

**Figure 2:**
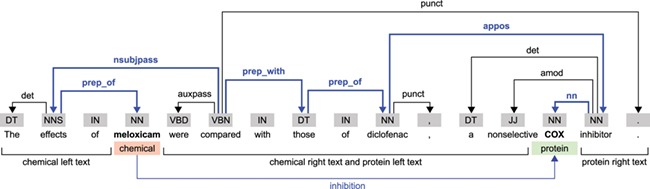
Example illustrating the dependency structure of a sentence from the CHEMPROT training dataset (PMID 10340919). In this example, we considered the relation between the ‘meloxicam’ chemical mention and the ‘COX’ protein mention. The SDP is highlighted in bold and blue color.

For every chemical–protein pair in each sentence, we obtain five sequences using the output of TEES: the SDP and the sequences containing the left text and the right text of the chemical and protein entities (Figure 2). Like the work of Mehryary *et al.* [29, 30], our system traverses the SDP always from the chemical entity to the protein entity. For entities spanning more than one word, we obtain the shortest path starting from the head word, as indicated by the TEES result. For each chemical–protein pair candidate instance, the chemical and protein entities (in cause) are replaced respectively by the placeholders ‘#chemical’ and ‘#gene’, except when the chemical–protein pair comes only from a single token (overlapped entities), which in this case is replaced by ‘#chemical#gene’. While in the SDP the dependency features were obtained traversing the path, in the four left and right sequences the incoming edge of each token was used as dependency features. If a token did not have an incoming edge or it was the last token in the SDP then the dependency feature was set to ‘#none’. Each one of the five sequences is therefore represented by a sequence of tokens, PoS tags and dependency edge labels.

Taking the sentence in Figure 2 as example, and considering the chemical–protein pair [‘meloxicam’, ‘COX’], the five extracted sequences (containing the tokens, PoS tags and dependency edges) are as follows:

1. **Shortest dependency path:** #chemical/NN/prep_of — effects/NNS/nsubjpass — compared/VBN/prep_with — those/DT/prep_of — diclofenac/NN/appos — inhibitor/NN/nn — #gene/NN/#none;
2. **Chemical left text:** The/DT/det — effects/NNS/nsubjpass — of/IN/#none;
3. **Chemical right text:** were/VBD/auxpass — compared/VBN/#none — with/IN/#none — those/DT/prep_with — of/IN/none — diclofenac/NN/prep_of —,/,/punct — a/DT/det — nonselective/JJ/amod;
4. **Protein left text:** in this case, it is the same as the chemical right text;
5. **Protein right text:** inhibitor/NN/appos —././punct.

The SDP together with the left and right sequences are fed to the neural network through embedding layers, as explained in the following subsections.

**Figure 3:**
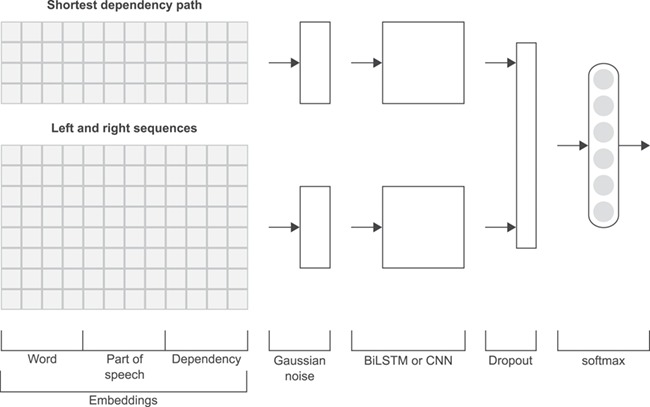
Neural network structure.

### Word embeddings

For text based tasks, it is necessary to encode the input data in a way that it can be used by the deep network classifier. This can be achieved by representing words as embedding vectors of a relatively small dimension, rather than using the large feature space resulting from the traditional one-hot encoding. Word embeddings is a technique that consists in deriving vector representations of words, such that words with similar semantics are represented by vectors that are close to one another in the vector space [51]. This way, each document is represented by a sequence of word vectors that are fed directly to the network. Efficient calculation of word embeddings, such as provided by word2vec [52], allow inferring word representations from large unannotated corpora.

We applied the word2vec implementation from the Gensim framework [53] to obtain word embeddings from 15 million PubMed abstracts in English language from the years 1900 to 2015. In previous research we created six models, with vector sizes of 100 and 300 features and windows of 5, 20 and 50. The models contain around 775 000 distinct words (stopwords were removed). These pre-trained word embeddings models showed their value achieving favorable results both in biomedical document triage [54] and biomedical WSD [55]. In this work we use the word embeddings models with a window size of 50, which are available in our online repository.

Another successor technique for creating word embeddings, from large unlabeled corpora, with subword information was proposed by Bojanowski *et al.* [56]. Their library, fastText, was used by Chen *et al.* [57] to create biomedical word embeddings (vector size of 200, and window of 20) from PubMed articles and MIMIC-III clinical notes [58]. We included these publicly available word embeddings in our simulations to compare to our own models.

Furthermore, we created PoS and dependency embeddings from the CHEMPROT dataset applying different vector sizes (20, 50, 100) and windows (3, 5, 10). The training, development and test sets are used, with 1020, 612 and 800 documents respectively (Table 1). However, we acknowledge the inclusion of the test set adds a slight bias. (A lapse that we do not find it worth for repeating all our simulations.) This could be overcome, possibly improving the overall results, by including (i) PubMed abstracts outside the CHEMPROT dataset or (ii) the remaining 2599 abstracts that initially existed, in the test set, to avoid manual annotations. Based on preliminary experiments on the training and development sets, we decide to use the pre-trained embedding vectors, with a window size of 3, which are kept fixed during training. We tested using randomly initialized PoS and dependency embeddings being adapted during training, but the results were similar and the runtime was higher.

Different tools (Gensim [53], fastText [56] and TEES [45]) were used for tokenization in the word embeddings creation and in the CHEMPROT dataset. Therefore, we created a mapping between the dataset vocabulary and its embedding vectors: each word of the CHEMPROT vocabulary was tokenized according to the word embeddings vocabulary, and its word vector was calculated using the L2-normalized sum of the constituent words. With this approach, the dataset vocabulary was strongly reduced (the respective PoS tags and dependency edges were also removed) because some uninformative tokens are not present in the word embeddings model. Preliminarily, this showed to be profitable since stopwords or out-of-vocabulary words were discarded from start.

We chose a fixed maximum length of 10 tokens (or 9 hops) for the SDP, and a maximum length of 20 tokens for each of the left and right sequences. These values were manually chosen according to the distribution of maximum lengths in the training set. We had tested using the length of the longer sequence, but this did not show to be advantageous since results were not better and implied a much higher training time. In the few cases in which the distance between the two entities is too long causing the extracted sequences to have more tokens than the pre-defined maximum, the sequences are truncated (the remaining tokens are discarded). In the opposite case, when there are less tokens than the maximum length allowed, the input vectors are padded with zeros to keep the same input vector size.

### Deep neural network

Figure 3 shows the general structure of the neural network used in this work. Similarly to other works in relation extraction [20, 25, 30], the different representations of a relation instance, namely the linear and SDP representations, are handled by two separate sub-networks, the results of which are concatenated at later stages.

Initially, each token in each one of the five extracted sequences (SDP, left and right texts) is represented by the concatenation of the embedding vectors from the word, PoS and dependency embedding matrices. Furthermore, the four left and right sequences corresponding to the linear representation are concatenated into a single input. For each of these two inputs (SDP and linear), Gaussian noise is added up, followed by a BiLSTM model or a CNN model (several convolution layers with multiple window sizes followed by global max pooling). Then, the two obtained outputs are concatenated and dropout is applied. At the final stage, a fully connected layer with softmax activation outputs the prediction probabilities. As can be seen in Figure 3, the neural network model only differs in an intermediate step (BiLSTM or CNN). We implemented these deep learning models in the Keras framework [59] and the TensorFlow backend [60] using the Python programming language [61].

An important consideration when defining and training deep network models is related to overfitting, which means that the network learns the ‘best’ data representation but is not able to generalize to new data. Various strategies have been proposed and are commonly employed to address this problem. In our experiments, we applied common strategies to avoid overfitting, namely random data augmentation (Gaussian noise addition), dropout and early stopping. Early stopping looks at the value of a specific evaluation metric in a validation subset and stops the training process when this value stops improving for a pre-specified number of training epochs (patience value). Also, early stopping brings a gain in total training time since the ‘best’ model is usually selected after a few epochs instead of training for a fixed, usually larger, number of epochs. This is an important aspect especially when running several simulations to test different network structures and parameters. According to preliminary results, we decided to fix 30% of the training data as validation subset, and calculated the F1-score at each epoch for monitoring the quality of the model. Similarly, when creating the final model to apply to the test data, we merged the training and development sets and used respectively 70% for training and 30% for validation and early stopping.

Table 2 shows the network hyper-parameters and other variables used in our system (default values were used in unmentioned parameters). Despite we did not perform an exhaustive grid-search for the best parameters, these were iteratively adjusted according to several experiments using the training and development sets. Class weights inversely proportional to their frequency in the training set were used to weight the input instances.

**Table 2:** System parameters

Gaussian noise standard deviation	0.01
LSTM units	128
LSTM recurrent dropout	0.4
LSTM dropout	0.4
Convolution filters	64
Convolution window sizes	[3, 4, 5]
Dropout rate	0.4
Optimizer	RMSprop [66]
Loss	Categorical cross entropy
Batch size	128
Maximum number of epochs	500
Early stopping patience	30
Early stopping monitor	Validation F1-score
Validation split	0.3

### Additional methods

To improve the generalization ability of our system and to reduce the fluctuation of the results due to the random initialization, all the results were obtained by averaging the prediction probabilities of three simulations using different random states. The use of a different random state means that a different random initialization was made in the neural network weights, and that distinct subsets of the training data were effectively used for training and validation.

Another crucial method in our system is the balancing between precision and recall to maximize the F1-score, achieved by adjusting the classification threshold at each training epoch. The training data is used in this process to avoid biasing the test results. A similar experiment was performed by Corbett *et al.* [28] where they also used a threshold value to maximize the F1-score on the development set.

Additionally, we pre-processed an external dataset from the BioGRID database [62] containing CPIs. This dataset supplied further 1102 PubMed abstracts for training, annotated with 2155 chemicals, 2190 proteins and 2277 relations between them.

In the next section we present and discuss the obtained results using the methods mentioned in this section.

## Results

As noted in the previous section, the use of different random states generates different training and validation subsets, which in turn results in different trained models (network weights and optimal classification threshold). This approach allows using a large amount of data for early stopping, which in our preliminary experiments proved important for improving generalization, while still using most of the available data for training. Thereby, the results presented in this section are obtained by averaging the probabilities from three simulations.

Table 3 presents a detailed gathering of results obtained on the development set by the BiLSTM and CNN models combining different inputs: sequences (SDP, left and right sequences), features (words, PoS, dependencies) and embedding models. The three best results on the development set (F1-scores: 0.6496, 0.6473 and 0.6385) were obtained by the BiLSTM model using only the SDP with word and dependency features where different embedding models are used, being the highest result achieved with the biomedical word embeddings created by Chen *et al.* [57].

**Table 3:** F1-score results on the CHEMPROT development set using the BiLSTM and CNN models. WS: word embeddings size. PS: part-of-speech embeddings size. DS: dependency embeddings size. SDP: shortest dependency path sequence. LR: left and right sequences. NN: neural network. W: words. P: part-of-speech tags. D: dependency edges. The highest value in each row is highlighted in bold; the best overall value is underlined

(WS, PS, DS)	Features	NN	W	P	D	W+P	W+D	P+D	W+P+D
(100, 20, 20)^a^	SDP	BiLSTM	0.6007	0.1695	0.2609	0.5971	**0.6385**	0.2991	0.6351
		CNN	0.5594	0.1628	0.2832	0.5622	0.5978	0.3102	**0.6010**
	LR	BiLSTM	0.4967	0.2003	0.2059	0.4906	**0.5149**	0.2106	0.5043
		CNN	**0.4371**	0.1902	0.1635	0.4131	0.4193	0.1683	0.3984
	SDP+LR	BiLSTM	0.5857	0.2271	0.3044	0.5776	**0.6000**	0.2807	0.5979
		CNN	0.5243	0.2332	0.2594	0.5268	0.5381	0.2361	**0.5403**
(300, 100, 100)^a^	SDP	BiLSTM	0.6161	0.1601	0.2920	0.6002	**0.6473**	0.3228	0.6310
		CNN	0.5642	0.1595	0.3019	0.5782	**0.6141**	0.2991	0.6092
	LR	BiLSTM	0.5135	0.2093	0.1910	0.5133	0.5209	0.1847	**0.5227**
		CNN	0.4293	0.1962	0.1550	**0.4576**	0.4321	0.1448	0.4216
	SDP+LR	BiLSTM	0.5914	0.2176	0.2873	0.5812	**0.6036**	0.2692	0.6015
		CNN	0.5572	0.2152	0.2519	0.5618	0.5672	0.2340	**0.5819**
(200, 50, 50)^b^	SDP	BiLSTM	0.6229	0.1530	0.2806	0.6192	**0.6496**	0.3087	0.6453
		CNN	0.5804	0.1555	0.2867	0.5841	**0.6259**	0.3182	0.6205
	LR	BiLSTM	0.5030	0.2353	0.2096	**0.5158**	0.5060	0.2166	0.4849
		CNN	**0.4813**	0.1827	0.1681	0.4504	0.4201	0.2130	0.4291
	SDP+LR	BiLSTM	0.5943	0.2428	0.2918	0.5993	**0.6126**	0.2715	0.5824
		CNN	0.5690	0.1966	0.2413	0.5440	**0.5760**	0.2645	0.5605

a ^a^Our PubMed-based word embeddings.

b ^b^Word embeddings by Chen *et al.* [57].

The results show that, in general, the left and right sequences generated much lower results, and when combining them with the SDP, the results were worse than using only the SDP. We believe this may be due to the way the left and right sequences are combined and encoded into the neural network, and also because the larger number of tokens (80 versus 10 in the SDP) may contribute with more noise by means of uninformative tokens. It is possible that different approaches for incorporating the linear sequence information could improve the final results.

As expected, words were the more informative type of feature, while the PoS tags were the less informative being worthless in some configurations. For example, in the majority of the cases, combining the PoS tags with words and dependencies worsened results. Interestingly, the dependency edge labels showed to be much more informative than the PoS tags, effectively improving performance in several configurations. Essentially, the highest results were achieved by combining words and dependency features.

Different embedding models were also explored (Table 3). We used larger embedding sizes for words, giving greater importance to word semantics, and smaller embedding sizes for PoS tags and dependency labels. The results show, in the case of our PubMed-based word2vec embeddings, that using larger encoding vectors ((300, 100, 100) versus (100, 20, 20)) leads to slightly improved results. Nonetheless, the best overall results were obtained with the fastText embeddings by Chen *et al.* [57], although these use a smaller vector size. This result highlights that the incorporation of subword information in the embedding vectors is beneficial for biomedical information extraction.

For collecting the final results (on the test set) we applied our described approach, but with two additional arrangements: (i) adding BioGRID external training data, and (ii) using no validation data (the validation split was set to 0.0). Table 4 presents these results using the best configuration based on the results obtained on the development set (Table 3), which consisted in using the SDP with word embeddings of size 200 (fastText model by Chen *et al.* [57]) and dependency features encoded by embedding vectors of size 50. For better comparison we also include in Table 4 the results of our best official run and the baseline results using our PubMed-based word embeddings.

**Table 4:** Detailed results on the CHEMPROT development and test sets using distinct approaches. The best configuration from the results in the development set (Table 3) was employed. WS: word embeddings size. PS: part-of-speech embeddings size. DS: dependency embeddings size. P: precision. R: recall. F: F1-score. The highest value in each column is highlighted in bold

			Development			Test		
(WS, PS, DS)			P	R	F	P	R	F
(300, 200, 300)^a^^,^^b^	Best official run		0.4999	0.6074	0.5470	0.5738	0.4722	0.5181
(300, 100, 100)^b^	Baseline^d^	BiLSTM	0.6737	0.6229	0.6473	0.7089	0.5480	0.6182
		CNN	0.7059	0.5435	0.6141	**0.7423**	0.4939	0.5932
(200, 50, 50)^c^	Baseline^d^	BiLSTM	0.6908	0.6130	**0.6496**	0.6812	0.5870	0.6306
		CNN	**0.7252**	0.5505	0.6259	0.7182	0.5093	0.5959
	BioGRID^e^	BiLSTM	0.5337	**0.6523**	0.5871	0.5881	**0.6050**	0.5964
		CNN	0.5913	0.5642	0.5774	0.6323	0.5191	0.5701
	No validation^f^	BiLSTM	0.6867	0.6068	0.6443	0.6791	0.5980	**0.6360**
		CNN	0.6247	0.4988	0.5547	0.6091	0.5160	0.5586

a ^a^Our official evaluated run [9, 10].

b ^b^Our PubMed-based word embeddings.

b ^c^Word embeddings by Chen *et al.* [57]

d ^b^Results on the development set are the same as reported in Table 3.

e ^e^30% of the training data (BioGRID excluded) used for validation.

b ^f^Model trained during 500 epochs (without monitoring).

**Table 5:** Comparison between participating teams in the CHEMPROT challenge (F1-score results on the test set)

Rank^a^	Work	Classifiers	Challenge	Post-challenge^b^
1	Peng *et al.* [24, 25]	SVM, CNN and RNN	**0.6410**	
2	Corbett *et al.* [27, 28]	RNN and CNN	0.6141	0.6258
3	Mehryary *et al.* [29, 30]	SVM and RNN	0.6099	0.6310
4	Lim *et al.* [31, 32]	Tree-structured RNN	0.5853	0.6410
5	Lung *et al.* [33, 34]	Traditional ML	0.5671	
6	Our work [10]	RNN and CNN	0.5181	0.6306
7	Liu *et al.* [35, 36]	CNN and attention-based RNN	0.4948	0.5270
8	Verga *et al.* [37]	Bi-affine attention network	0.4582	
9	Wang *et al.* [67]	RNN	0.3839	
10	Tripodi *et al.* [68]	Traditional ML and neural networks	0.3700	
11	Warikoo *et al.* [44, 69]	Tree kernel	0.3092	0.3654
12	Sun [9]		0.2195	
13	Yüksel *et al.* [70]	CNN	0.1864	

a ^a^Teams ranked according to the official evaluation.

b ^b^Improved results due to post-challenge enhancements.

**Table 6:** Confusion matrix in the CHEMPROT test set (F1-score 0.6306) obtained by the BiLSTM model that achieved the highest F1-score in the development set, as reported in Table 4. Green cells show correct classifications (true positives); pink cells show false positives; yellow cells show false negatives (first line) and misclassifications between classes. Differences to the best results obtained during the challenge are shown in parentheses

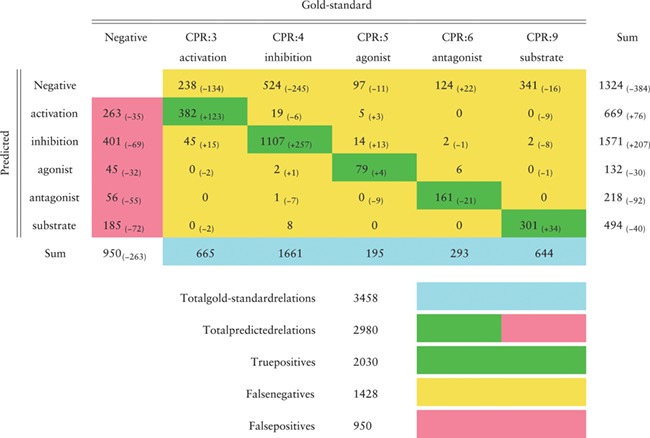

**Table 7:** Heatmap representing the precision values obtained by the BiLSTM model (the best in the development set) applied to the CHEMPROT test set. True positives (TP) and false positives (FP) are displayed as $\frac {\textit {TP}}{\textit {FP}}$. X-axis: number of gold-standard entities per sentence. Y-axis: number of gold-standard evaluated relations per sentence. Axes are truncated for conciseness. GS: gold-standard

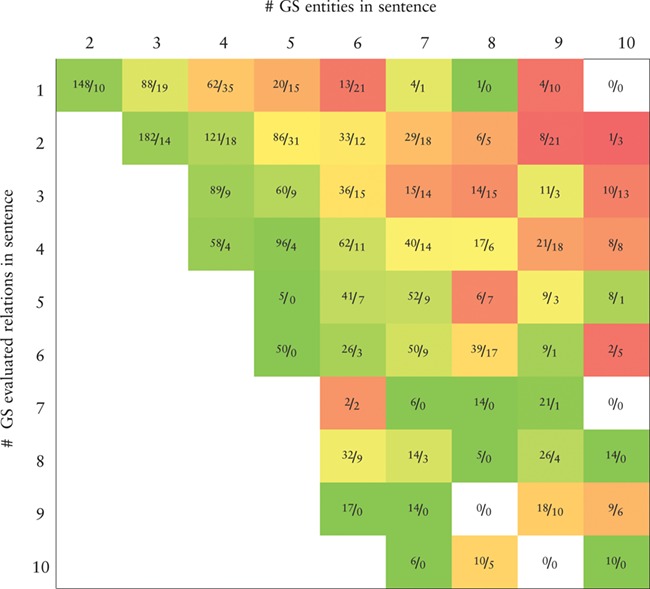

Inclusion of the BioGRID dataset as additional training data deteriorated F1-score results when compared to not using it, in both BiLSTM (development: 0.5871 versus 0.6496, test: 0.5964 versus 0.6306) and CNN models (development: 0.5774 versus 0.6259, test: 0.5701 versus 0.5959). This suggests that these data diverge from the CHEMPROT guidelines and that some kind of heuristics would be required to decide which instances to include. Other approaches such as multi-instance [63] or adversarial learning [64] could also be applied.

Inspection of the training and validation F1-score for each epoch indicates that the BiLSTM model suffered less from overfitting than the CNN model. Therefore, we performed an experiment where models were trained for 500 epochs without early stopping, since this has the advantage of training each model (in the three simulations) using all the available training data. Overall, the highest F1-score on the test set was achieved following this approach (0.6360 versus 0.6306 in the baseline) showing that the BiLSTM model was in fact very resistant to overfitting. Conversely, the CNN performed much worst when early stopping, and therefore validation data, was not used (0.5586 versus 0.5959). Even when trained with the BioGRID external dataset, where validation data was used, the CNN model obtained better results compared to those obtained without validation monitoring (0.5701 versus 0.5586). Despite 0.6360 being the highest F1-score in the test set, we consider our best F-score is 0.6306 since it is selected according to the best method in the development set (Table 4), which represents an improvement of 11 percentage points compared to our best official F1-score (0.5181).

**Table 8:** Heatmap representing the recall values obtained by the BiLSTM model (the best in the development set) applied to the CHEMPROT test set. True positives (TP) and false negatives (FN) are displayed as $\frac {\textit {TP}}{\textit {FN}}$. X-axis: number of gold-standard entities per sentence. Y-axis: number of gold-standard evaluated relations per sentence. Axes are truncated for conciseness. GS: gold-standard

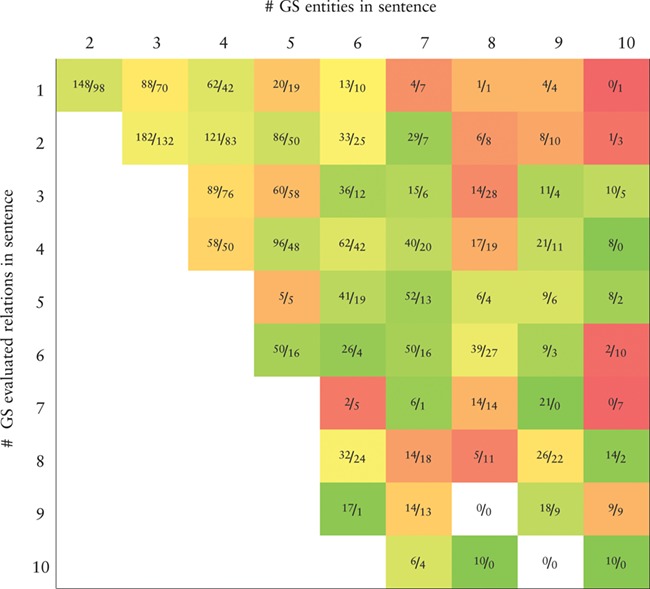

**Table 9:** Error analysis: examples of incorrect predictions in the CHEMPROT test set obtained by the BiLSTM model (the best in the development set). The chemical–protein pairs are presented with information from the sentence and the shortest dependency path (SDP). The chemical and protein named entities are shown in italic and annotated with the [chemical] and [gene] tags. For simplicity, the chemical and gene placeholders were omitted in the list of words from the SDP.

Example	Correct	Predicted	Full sentence	Words in the SDP
1	Agonist	Activation	The introduction of the *amino*$_{\text {[chemical]}}$ group resulted in not only improved water solubility but also enhanced *TLR7*$_{\text {[gene]}}$ agonistic activity.	Group introduction resulted activity
2	Agonist	Inhibition	Our work shows that *sulfonylureas*$_{\text {[chemical]}}$ and glinides additionally bind to PPARgamma and exhibit *PPARgamma*$_{\text {[gene]}}$ agonistic activity.	Exhibit activity
3	Antagonist	Agonist	In guinea pigs, antagonist actions of *yohimbine*$_{\text {[chemical]}}$ at *5-HT(1B)*$_{\text {[gene]}}$ receptors are revealed by blockade of hypothermia evoked by the 5-HT(1B) agonist, GR46,611.	Receptors
4	Activation	Inhibition	Impaired expression of the uncoupling protein-3 gene in skeletal muscle during lactation: fibrates and *troglitazone*$_{\text {[chemical]}}$ reverse lactation-induced downregulation of the *uncoupling protein-3*$_{\text {[gene]}}$ gene.	Reverse downregulation gene
5	Inhibition	Activation	*Geldanamycin* $_{\text {[chemical]}}$ also disrupts the T-cell receptor-mediated activation of *nuclear factor of activated T-cells*$_{\text {[gene]}}$ (NF-AT).	Disrupts activation
6	Substrate	Inhibition	Blockade of *LTC4*$_{\text {[chemical]}}$ synthesis caused by additive inhibition of *gIV-PLA2*$_{\text {[gene]}}$ phosphorylation: effect of salmeterol and PDE4 inhibition in human eosinophils.	Synthesis caused inhibition phosphorylation

From the results in Tables 3 and 4, we conclude that a solid benefit of our approach is that the best method uses at most 10 tokens from the SDP to classify the CPR, using a small representation vector and therefore reducing training time. For instance, on an Intel i3-4160T (dual-core, 3.10 GHz) CPU, training the BiLSTM and CNN models for one epoch with 70% of the training set (word and dependency embeddings with sizes 100 and 20) takes respectively around 5 and 2 seconds (the additional cost of balancing precision and recall is excluded). Also, another positive remark is that our BiLSTM model is resistant to overfitting, since the results obtained in the baseline approach are similar to those reported without using validation data, and the results in the development and test sets are similar. On the other hand, overfitting is evident when using the CNN model, since training it for 500 epochs grossly declined the results (development: 0.6259 versus 0.5547, test: 0.5959 versus 0.5586). This overfitting also helps explain the higher precision seen for the CNN model as compared to the BiLSTM model, since the network is better capable of identifying with high confidence those test instances that are very similar to instances seen during training.

### Comparison with other participating teams

Table 5 compares our results with other works presented during the CHEMPROT challenge as well as post-challenge improvements. All the top performing teams used RNNs showing their strength in this CPR extraction task. Also, SVMs and CNNs are among some of the classifiers used by other works.

Similarly to our work, Corbett *et al.* [26, 28] used LSTM and CNN layers. They achieved a best F1-score of 0.6258 on the test set, which is in line with our result (0.6306). However, their network structure is larger being composed of more layers. Mehryary *et al.* [29] applied a similar pre-processing pipeline as described in this work, using the TEES tool to perform tokenization, PoS tagging and dependency parsing. They achieved a top F1-score of 0.6099 with a combination of SVMs and LSTM networks. This result was improved to 0.6310 following the challenge [30]. Using the ANN alone, with whole sentence tokens and features from the SDP, they achieved an F1-score of 0.6001 in the test set, while our BiLSTM model achieves an F1-score of 0.6306 by only using features from the SDP. Lim *et al.* used a tree-structured RNN exploiting syntactic parse information [31, 32] and obtained an F1-score of 0.6410, equalling the best official result.

Differently from the works cited above, Lung *et al.* [34] used traditional machine learning algorithms with handcrafted features, achieving an F1-score of 0.5671. As part of their approach, the authors manually built a dictionary with 1155 interaction words, which where mapped to the corresponding CPR type, to create CPI triplets.

## Discussion

In this section we evaluate, making a detailed error analysis, the predictions obtained in the test set using the baseline approach with the fastText word embeddings and the BiLSTM model (Tables 6, 7, 8 and 9). The confusion matrix, presented in Table 6, follows the official evaluation script and reflects the same results reported in Table 4. The improvements in comparison to our best official run are also indicated, showing that our current system predicted more correct cases except for the ‘antagonist’ relation class where 21 more cases were missed. The number of false positives was significantly reduced for all the classes, while the number of false negatives diminished overall but increased for the ‘antagonist’ relation class. The ‘activation’ and ‘inhibition’ relation classes were the ones most difficult to discriminate, with 19 ‘inhibition’ relations predicted as ‘activation’ and 45 ‘activation’ relations predicted as ‘inhibition’.

Tables 7 and 8 show, respectively, heatmaps of the precision and recall values in function of the numbers of gold-standard entities per sentence and gold-standard relations per sentence. Numbers in the cells show the amount of correct classifications (true positives) and incorrect (false positives) or missed classifications (false negatives). This representation makes it easier to understand which type of sentences are more difficult for our model to ‘interpret’. In Table 7 we see a clear and somewhat expected trend with lower precision when the number of entities in a sentence is high but the number of existing relations in that sentence is low. This is intuitive since many chemical–protein pair candidates are generated, potentially leading to several false positive relations. From Table 8 we verify that the majority of the sentences in the corpus have only a few number of entities and relations. Sentences with many entities are rare, and these may have few or many relations. Interestingly, the results in Table 8 indicate that, although the worst results in terms of recall are obtained for sentences containing many entities, there is a considerable number of unidentified relations from sentences containing up to four entities.

### Error analysis

We present a detailed error analysis showing concrete cases where the model failed to predict (Table 9). A comprehensive list with all the predictions can be found in the online repository. We enumerate different causes for the analyzed frequent errors:

1. Limited or incorrect instance representation. Information obtained exclusively from the SDP is, often, insufficient or faulty since essential words may be lacking or misleading words may be present. Examples 1, 2 and 3 in Table 9 show cases where crucial terms such as ‘agonistic’ and ‘antagonist’ are not included in the SDP. On the other hand, examples 4, 5, 6 include words, such as ‘downregulation’, ‘activation’ and ‘inhibition’, that are frequently related with other relation classes and caused incorrect classification in these cases.
2. Misinterpretation of negation. In some cases, there is a term giving the opposite meaning to the textual sequence. However, these terms are not correctly handled by our model. For example, cases 4 and 5 have, in the SDP, the expressions ‘reverse downregulation’ and ‘disrupts activation’, which should direct to the true relation classes, namely activation and inhibition.
3. Complex sentences, requiring expert interpretation. Some cases, as in example 6, are not easily interpreted without domain knowledge or more context.

To counteract these errors, we hypothesize that improved feature representations and more training data may alleviate these issues. Also, we suspect that building a system for multi-label classification would improve recall, and could improve the final results, since there are failed predicted relations that count simultaneously as a false positive and a false negative.

Another limitation of our model is that for each chemical–protein pair only information from the respective sentence is being used. We suspect more context would prove helpful, and could facilitate possibility of extraction of cross-sentence relations.

## Conclusions and future work

This paper describes neural network architectures for CPI extraction and the improvements we accomplished following our participation in the CHEMPROT task of the BioCreative VI challenge (Track 5). Our methods consist of using deep learning classifiers with input features encoded by embedding vectors. We use word embeddings pre-trained in biomedical data, while PoS and dependency embeddings were pre-trained from the CHEMPROT dataset. Our best proposed models, BiLSTM and CNN, achieved top F-scores of 0.6306 and 0.5959 on the test set, respectively. The BiLSTM model showed its convenience being more resistant to overfitting than the CNN model.

We mapped CPIs from the BioGRID interaction repository to CHEMPROT classes, to add as additional training data. However, inclusion of these data did not improve results, and we believe that a more accurate handling of these data could prove effective. The use of other external resources such as knowledge bases, datasets or repositories should also be considered.

Although we applied these methods to relations between chemical and protein entities, the methods are general and can be applied to any relation type for which a training corpus is available. As such, as future work we aim to apply a similar approach for extracting different biomedical relations such as drug–drug, PPIs and chemical–disease relations. Additionally, we are interested in exploring different network architectures such as tree-structured networks [65], hierarchical networks [20, 40] and attention mechanisms [38, 39].

## Database URL

https://github.com/ruiantunes/biocreative-vi-track-5-chemprot/
